# Surgery.—Hernia

**Published:** 1904-06-11

**Authors:** 


### SURGERY.—HERNIA.

(*Continued from page* 174.)

**Two Cases of Retroperitoneal Hernia.**—Mitchell[1] reports these cases which both belonged to that variety which occurs into the duodenal fossæ. Case 1, a woman aged 30 who was shot through the heart dying immediately. At the autopsy, instead of coming down on the intestines after opening the abdomen a white glistening sac was found which was discovered to be a retroperitoneal hernia. It was situated in the centre and left side of the abdomen surrounded by the colon and extending below to near the promontory of the sacrum. At the lower aspect was an elliptical opening from which the lower ileum escaped to join the cæcum. The sac was smooth and shining, the walls thick but translucent. The orifice was situated low down close to the cæcum and looked forward and to the right. The upper margin was thick, the inferior mesenteric vein bordered the opening and the colica sinistra ran at some little distance from the free border. The coils of intestine in the sac were patent and moderately distended with gas but were strongly adherent so that only part of them could be pulled out. According to Treitz three things are essential for the formation of this variety of hernia. (1) A dilatable excavation of the peritoneum furnished by the fossa duodeno-jejunalis, (2) a resisting ring furnished by the vascular arcade composed of the inferior mesenteric vein and the colica sinistra artery,[4] and (3) a mobile intestine. Case 2, a child of who died of burns. At the autopsy the small intestine was seen to lie in a separate peritoneal pouch behind the general peritoneal cavity. The opening of this was as large as the palm of the hand with thickened, smooth edges. The opening seemed to be on the right side; the whole of the jejunum and ileum lay within this sac. The head of the cæcum and appendix lay outside it free and somewhat raised above the level of the right iliac fossa.

**Richter's Hernia.**—Two cases are reported from the transactions of the Philadelphia Academy of Surgery.[5] In the first a woman aged 42 was taken with severe vomiting following an over-dose of an expectorant mixture. Whilst straining, she felt a sharp colicky pain over the whole abdomen. The pain continued, and the bowels did not act although purgatives were given. On the fourth day the abdomen had begun to swell, but as the vomiting had ceased, intestinal obstruction was not suspected. No hernia could be found, and the patient insisted that she had never had one. On the seventh day vomiting commenced again, and was fæcal, and on the next day there were evidences of general peritonitis. Operation was performed, an incision being made below the umbilicus, and on carrying the hand to the right iliac region, a portion of intestine was found fixed in the right femoral canal. This was detached, and the portion of bowel delivered presented the appearance of a large nipple, and was gangrenous. A resection of the bowel was performed, but the patient died of general peritonitis. The second case was a man aged 68 who had a large phlegmon of the right groin. Twelve years previously he had "an enlarged gland" in the right groin, which disappeared, and was very probably a hernia. Four weeks before coming under observation he was seized with griping pains in the abdomen and in the meantime he observed an enlargement in the right groin which took on growth and steadily increased. The skin was dusky and there was throbbing pain and fluctuation. On incising the abscess, 10 ozs. of pus and bowel contents escaped. At the site of the femoral canal there was a small opening which communicated with the bowel. A fæcal fistula remained, but six months later a resection and end-to-end anastomosis was made and was followed by recovery.

**Diaphragmatic Hernia.**—Chadbourne[6] reports the case of an epileptic aged 30 who soon after dinner vomited, became short of breath, and complained of pain in the abdomen and had great abdominal distension. His bowels acted once after the onset of the symptoms, and he died on the second day. At the autopsy there was enormous distension of the abdomen. There was a large defect in the left half of the diaphragm through which had passed the stomach, the omentum, and the large intestine from three inches above the cuput cæcum and including a portion of the descending colon. No obstruction other than the hernial ring could be found. The heart was displaced to the right. After allowing the escape of the gas the displaced viscera could be replaced without much difficulty, there being no adhesions. A second case[7] is recorded of a man aged 61 who was suddenly seized with intense epigastric pain and vomiting. He passed neither flatus nor fæces for three days. After the application of poultices to his abdomen, his pain was relieved and his bowels acted, so no operation was performed. There was a history of two previous attacks lasting several days. On examination the epigastric region was protuberant and dome-like, contrasting markedly with the flatness of the abdomen below the umbilicus. The lower part of the thorax on the left side was enlarged. On percussion the protuberant epigastrium and left infra-mammary region gave a note like the sound of a kettle-drum. The liver dulness was diminished and the heart apex in the fourth intercostal space. Metallic sounds were heard all over the resonant area and on auscultation no breath sounds could be heard over the lower part of the thorax corresponding to the drum-like percussion area, but on deep respiration amphoric reverberations were heard not only over this part of the thorax but also over the distended epigastrium. Gurgling and cooing sounds were also heard occasionally. As regards diagnosis the drum-like sound, the metallic phenomena, and the absence of normal lung sounds pointed to the presence of a hollow space filled with gas or air in the lower part of the thorax continuous with the hollow space in the epigastrium. The bowel noises in this space excluded an ordinary pneumothorax and suggested that a distended portion of the alimentary canal had entered the left pleural cavity. The patient was discharged with his symptoms of obstruction relieved, but the hernia into the pleural cavity practically unchanged.

[4] Amer. Jour. of Med. Scien., Nov. 1903. [5] Annals of Surg., March, 1904. [6] Amer. Jour. of Med. Scien., Sept. 1903. [7] Med. Chron., April, 1904.

*(To be continued.)*

